# Supplementary figures and images for: In pursuit of visual attention: SSVEP frequency-tagging moving targets
Source: PLoS One. 2020 Aug 4;15(8):e0236967. doi: 10.1371/journal.pone.0236967 (PMC7402507; doi:10.1371/journal.pone.0236967)

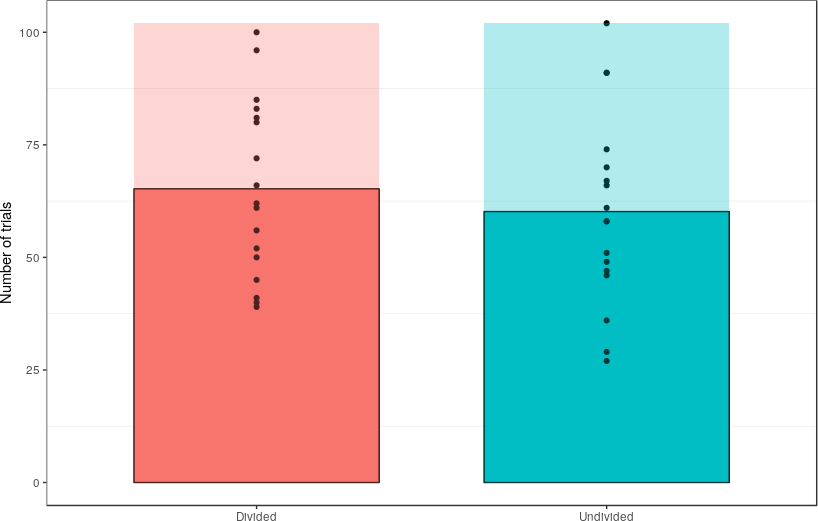

Supplement: S1 Fig — Trials were excluded due to unreliable tracking of the targets in both conditions from a total of 102 presented in each condition. Dots represent accepted trial numbers for each participant. (TIF) [file pone.0236967.s001.tif]

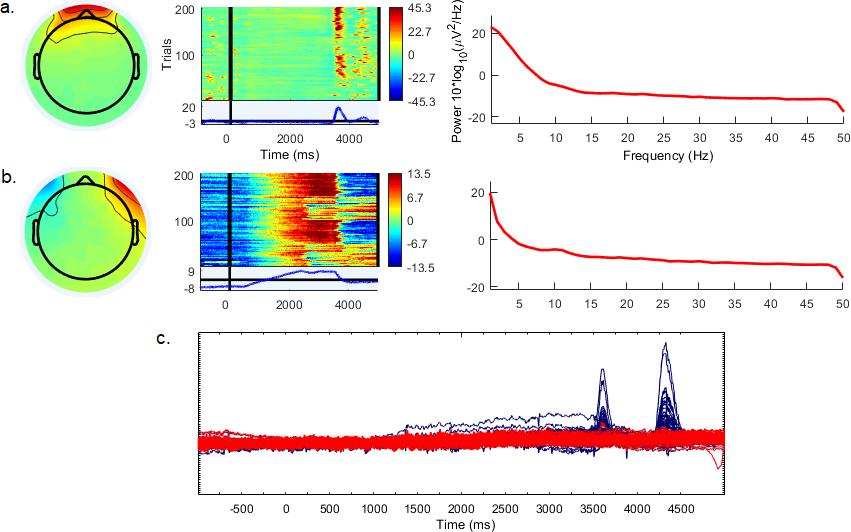

Supplement: S2 Fig — Distortions isolated and removed through the ICA process encompassed (a.) eye-blinks, and (b.) eye-movements involving saccades (sharp onset/offset activity), as well as smooth pursuit (slow drift). Fig 2C represents a single-trial example of uncorrected raw EEG (blue) and ICA corrected EEG (blinks, saccades, and drift removed) (red). (TIF) [file pone.0236967.s002.tif]
